# Patient–Health Care Professional Communication via a Secure Web-Based Portal in Severe Mental Health Conditions: Qualitative Analysis of Secure Messages

**DOI:** 10.2196/63713

**Published:** 2025-06-27

**Authors:** Eva Meier-Diedrich, Carolyn Turvey, Jonas Maximilian Wördemann, Justin Speck, Mareike Weibezahl, Julian Schwarz

**Affiliations:** 1 Department of Psychiatry and Psychotherapy, Center for Mental Health Immanuel Hospital Rüdersdorf Brandenburg Medical School Theodor Fontane Rüdersdorf Germany; 2 Faculty of Health Sciences Brandenburg Brandenburg Medical School Theodor Fontane Neuruppin Germany; 3 Center for Access & Delivery Research and Evaluation Iowa City Veterans Affairs Health Care System Iowa City, IA United States; 4 Office of Rural Health Veterans Rural Health Resource Center – Iowa City Iowa City Veterans Affairs Health Care System Iowa City, IA United States; 5 Department of Psychiatry University of Iowa Iowa City, IA United States; 6 Center for Health Service Research Brandenburg Brandenburg Medical School Theodor Fontane Rüdersdorf Germany

**Keywords:** secure messaging, open notes, online record access, ORA, psychiatry, eHealth, mobile health, mHealth

## Abstract

**Background:**

Patients’ web-based access to their medical records and secure messaging (SM) via patient portals is becoming increasingly prevalent worldwide. SM offers several potential benefits, including improved health outcomes and increased patient engagement. However, SM also raises concerns about effects on the therapeutic relationship and may be constrained by factors such as limited digital literacy and access to digital devices. Evidence on the use of SM in mental health is limited, and results are inconclusive.

**Objective:**

This study aimed to examine (1) the purposes for which health care professionals (HCPs) and patients with psychiatric disorders use SM to communicate and (2) the specific use patterns associated with both patients and HCPs.

**Methods:**

The secure messages (n=274) of 38 patients with psychiatric disorders and 4 HCPs (psychiatrists) from 3 psychiatric outpatient clinics in Brandenburg, Germany, was analyzed using thematic analysis. The data selected for this study represent a subsample from a larger study comprising a total of 116 patients. The subsample consists of the patients and HCPs who used SM.

**Results:**

A total of 274 messages were analyzed: 22.3% (61/274) were initial notes from HCPs, 44.5% (122/274) were patient responses, and 33.2% (91/274) were HCP replies. Patients sent between 1 and 15 messages (mean 4.16, SD 3.42) and logged in 1 to 42 times (mean 10.78, SD 9.38). Most messages were sent during the day, although some were also sent at night and in the early morning. Regarding the purposes of SM, 4 core functions of SM were identified: reporting and feedback, interpersonal uses, intrapersonal uses, and organizational uses. Both patients and HCPs used SM to share treatment-relevant information and elicited feedback on treatment and medication. Furthermore, secure messages included expressions of gratitude by the patients, in addition to well-wishes and emotional support from the HCPs. SM allowed patients to reflect on their treatment and provide self-encouragement. Finally, secure messages were used to address organizational aspects such as scheduling, appointments, and administrative tasks.

**Conclusions:**

SM in outpatient mental health care is multifaceted and holds the potential to enhance therapeutic contact and improve access to care by enabling quick, low-threshold communication between patients and HCPs, allowing treatment-related concerns to be addressed promptly and effectively. However, the asynchronous nature of SM also poses new challenges, particularly in managing acute mental health crises and in setting boundaries to prevent HCPs from being perceived as constantly available. Therefore, specific training for HCPs—both during medical education and in clinical practice—is essential, along with clear guidelines on handling crises and managing sensitive information.

## Introduction

### Background

With a growing number of countries worldwide allowing web-based medical record access to patients, the integration of secure messaging (SM) systems into patient portals is also increasing [1]. SM is recognized as a valuable tool with multiple benefits in health care. It is one of the most popular features of patient portals among both patients and health care professionals (HCPs) [2] and has been rapidly adopted in recent years [3,4]. SM enables patients and HCPs to communicate effectively, provide treatment feedback, and address urgent questions without the need for them to be postponed until the next in-office visit [5]. Therefore, SM serves as a preventive measure against treatment delays caused by long wait times [6], which can be particularly prominent in the field of mental health [7].

Providing patients with access to a patient portal is already well established internationally, particularly in the United States and the northern European countries [8]. However, the introduction of an SM feature is a relatively recent development in the field. In Sweden, patients were previously able to comment on their electronic health records (EHRs) [9], and while this feature was well accepted and frequently used by patients, it was discontinued in 2022 due to technical issues [10]. In Finland, patients can save data to their personal health record through certain (limited) well-being applications; however, HCPs currently do not have access to the data [11]. Similarly, in Germany, the need for an effective and secure digital communication tool in health care has been recognized, leading to the enactment of various laws in recent years, such as the “Act for Secure Digital Communication and Applications in Healthcare” (E-Health Act). This legislation paved the way for the mandatory implementation of an EHR for all patients by 2025 (so-called opt-out). In addition, the Hospital Future Act, which came into force in Germany in 2020, mandates the implementation of patient portals, including (asynchronous) communication between patients and HCPs [12].

Studies indicate that SM via patient portals can facilitate access to care [13], enhance patient-clinician communication, and enable efficient and location-independent information exchange between patients and HCPs [5,14]. This appears to be of particular importance in psychiatric systems, where therapeutic contacts are often brief and infrequent—typically 1 appointment every 3 months lasting 7.5 to 30 minutes for patients affected by mental health conditions. Furthermore, SM has been associated with improved health outcomes [15], greater patient engagement (eg, better treatment adherence [16], higher clinic attendance [17], and improved disease management and disease awareness [18]), and increased patient satisfaction [13]. Reported effects of SM on the frequency of in-office visits remain inconclusive [3-5,19]. SM is commonly used by patients for self-management of chronic diseases, accessing preventive services, and communicating with HCPs (eg, to inquire about symptoms or laboratory results, request prescription refills, or schedule appointments) [15,20]. Both patients and HCPs are generally in favor of using SM [14]. Patients particularly value the speed, ease, and directness of communicating through SM, perceiving it as an extension—and sometimes even replacement—of the in-office treatment [5,20]. Similarly, HCPs generally have a positive view of SM, appreciating its usefulness for tasks such as prescription refills and patient monitoring [14,20].

Despite its many advantages, both patients and HCPs have some concerns. Patients worry about the loss of interpersonal contact [21], the appropriate manner of engagement [14], and the possibility of taking up too much of their clinicians’ (uncompensated) time [5,14]. Additional patient use barriers include time constraints, limited usability and patient portal accessibility, and lack of digital literacy [5]. The privacy, security, and governance of sensitive health data have been a major concern for both parties [22]. HCPs encounter new demands in the practical implementation, such as potential institutional and workflow barriers (uncompensated hours and poor design), which could result in an additional workload [20,23] and the pressure to respond immediately [14,20]. HCPs express concerns that patients may misinterpret the proper use of the messaging feature [14] and already face difficulties in managing certain “high-demand” patient messages due to their length, frequency, urgency, complexity, or lack of clarity [14,20].

### Objectives

There is only limited evidence on patient portals in mental health [24-28]. Studies on SM through patient portals in mental health are even fewer and vary depending on the outcome measures. While there is some indication of increased engagement through SM [29], the effects on medication adherence remain limited [30]. Nevertheless, both patients and HCPs in the mental health care field seem to welcome the introduction of SM [20,31]. Evidently, the implementation of SM via patient portals in mental health needs further exploration [20,29]. Therefore, this study aims to thoroughly explore the use of the SM function in patient portals by patients affected by a mental health condition. The following research questions will be addressed: (1) for what purposes do HCPs and patients affected by a severe mental health condition use SM to communicate in the outpatient treatment setting? and (2) what characterizes the use patterns of HCPs and patients?

## Methods

### Design

This study is part of the PEPPPSY (Piloting and Evaluation of Participatory Patient-Accessible Electronic Health Record in Psychiatry and Somatics) project (since 2021), which centers on piloting and evaluating a participatory patient record in psychiatry and somatic medicine [32,33]. The main objective is to investigate the development, implementation, processes, and outcomes of the corresponding patient portal, referred to as PEPPPSY, from the perspectives of patients and HCPs. Following the exploratory nature of this study, a qualitative method was chosen to comprehensively analyze the use of SM through patient portals.

### PEPPPSY App

**Figure 1:**
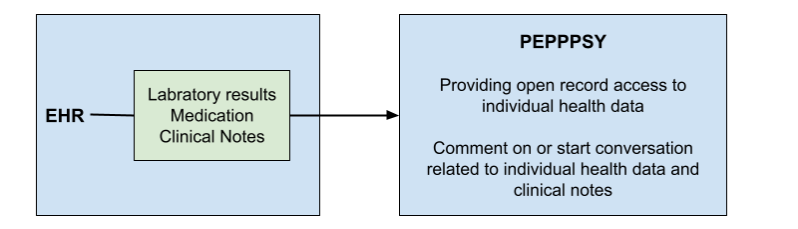
Relationship between the electronic health record (EHR) and the web-based patient portal PEPPPSY (Piloting and Evaluation of Participatory Patient-Accessible Electronic Health Record in Psychiatry and Somatics) and its functions.

The pilot patient portal PEPPPSY was created through a research collaboration between the Norwegian University of Science and Technology and the Brandenburg Medical School. PEPPPSY is a stand-alone web-based technology designed to facilitate access to specific elements of a (digital) medical health record for research purposes. PEPPPSY is a patient portal that accesses data from the EHR and extends it with additional functions, as illustrated in Figure 1.

PEPPPSY is the result of an (ongoing) iterative process of participatory design, development, application, and evaluation [32]. PEPPPSY’s primary function is to provide patients with secure access to view their clinicians’ clinical notes. The practice of sharing clinical notes with patients is usually described with the term Open Notes. The pilot does not yet have a messaging function separate from the clinical notes. Nevertheless, patients have the possibility to respond to each clinical note and initiate SM with their HCPs. It is not uncommon for patients to use this SM option, independently of the initial clinical notes, both in terms of time and content, to contact their HCPs. In the patient portal settings, patients and HCPs could configure email notifications for new messages in the patient portal. HCPs had no specific requirements regarding how quickly they needed to respond or the time of day responses should be sent. Table 1 illustrates a fictional example of a messaging interaction via PEPPPSY that closely resembles actual practice. In the results, each quote is linked to a specific HCP (HCPXX) or patient (PTXX) using an abbreviation followed by a number. In the current second phase of the pilot, PEPPPSY is being expanded to serve a broader patient population, offering additional features such as the SM function or a proxy access for care partners.

**Table 1:** Example of messaging via the patient portal, including an initial clinical note by a health care professional (HCP), the patient’s response, and the HCP’s reply.

Sequence of the interaction	Excerpt from fictional secure message
Initial clinical note by an HCP	“The dosage of venlafaxine was decreased gradually from 150 mg to 75 mg due to the patient’s report of excessive perspiration.” [HCPXX]
Patient’s response	“Unfortunately, the medication hasn’t helped. I’ve been trying 75mg for two weeks now, but I’m still sweating. What can we do?” [PTXX]
HCP’s response	“Thank you for your message. Please come to my office. We should reduce or discontinue the current medication and then switch to a different one.” [HCPXX]

### Ethical Considerations

Ethics approval was obtained from the Ethics Committee of the Brandenburg Medical School (E-01-20210727), and the study was registered with the German Clinical Trial Register (DRKS00030188). All data collected were deidentified. Within the SM, names, age, family relationships, location, gender, and times were randomly pseudonymized. The participating HCPs received a single compensation payment of €50 (US $57) for their participation in the study.

### Study Setting

The study was conducted at 3 psychiatric outpatient university clinics of Immanuel Hospital Rüdersdorf, part of Brandenburg Medical School. Located in the Berlin/Brandenburg metropolitan region, these clinics provide mental health care services to approximately 255,000 residents in the catchment area. These outpatient clinics are dedicated to offer specialized mental health care for patients who need a comprehensive and multidisciplinary approach, considering the nature, severity, or duration of their condition. There are approximately 500 psychiatric outpatient clinics (POCs) in Germany, treating approximately 2 million patients a year [34]. Treatment eligibility in respective clinics is based on specific diagnoses—including severe mental illness and other approved conditions—as determined by insurance providers and the hospital association [35]. The implemented multidisciplinary team approach includes psychiatrists, psychotherapists, nurses, and social workers.

### Recruitment and Sampling

The inclusion period spanned from October 2022 to February 2024. For this branch of the study, all patients who used the SM function between December 2022 and June 2023 were included. Consequently, this study exclusively comprises HCPs whose patients had sent at least 1 secure message to them.

For the PEPPPSY study, patients were recruited using a purposive sampling strategy, targeting individuals who met the following inclusion criteria: a minimum age of 18 years; a diagnosis of severe mental illness based on the German version of the *International Classification of Diseases, Tenth Revision* (*ICD-10*) criteria; ongoing outpatient treatment in one of the study centers within the last 6 months; and sufficient capacity to provide informed consent for the study. Qualified HCPs (eg, treating psychiatrists or psychotherapists) assessed suitability for participation. Patients with suicidal tendencies, patients who posed a danger to others, as well as patients affected by psychosis or severe cognitive impairment regardless of their diagnosis were excluded from participation in the study. Eligible patients were approached face-to-face by their HCPs and, if interested, were provided with information about the study’s objectives and procedures. Those who agreed to participate provided written informed consent. Patients were then introduced to the patient portal by their HCP or a study team member and granted access. They also received a written user manual, which included detailed step-by-step instructions and screenshots. Study team members were available to assist both patients and HCPs with any technical or other questions. Typically, patients accessed the patient portal via their personal digital devices (eg, smartphones or laptops). If patients lacked such devices, they could use a study tablet at the study center to access the portal. However, all participating patients were required to have a device capable of receiving SMS text messages, as this was essential for secure 2-factor authentication to log into the patient portal.

HCPs at the study centers were contacted by the study team and informed about the PEPPPSY study’s objective, procedures, and compensation (€50 per HCP). Participating HCPs provided informed consent and were introduced to the patient portal by a member of the study team. HCPs typically accessed the patient portal using their professional computers and professional mobile devices. To be eligible, they had to be older than 18 years, capable of providing informed consent, and employed at one of the study centers.

### Data Collection

All participants were informed about the study both verbally and in writing and provided written informed consent. Sociodemographic data were collected. All patients and HCPs were able to contact the study team (patients could also reach out directly to their HCPs for support) at any time if they had technical problems with the patient portal.

All SM between HCPs and patients from December 2022 to July 2023 was retrieved from the PEPPPSY patient portal and stored in a tabular format. For improved clarity and readability, the term messages will consistently be used throughout the paper.

### Data Analysis

Thematic analysis of the SM was conducted using the MAXQDA software (VERBI GmbH) [36]. A category system was devised to examine the use patterns of SM exchanged between patients and HCPs. Initially, one-third of the retrieved messages were analyzed deductively, and then, the analysis was extended to include inductive categories emerging from the data [37]. Two researchers performed the initial analysis and ensured consensus. When consensus could not be reached, a third researcher joined the analysis. In addition, participants’ sociodemographic data were analyzed based on their messaging behavior. The COREQ (Consolidated Criteria for Reporting Qualitative Research) checklist was used for quality assurance (Multimedia Appendix 1).

## Results

### Patient Characteristics

A total of 123 patients were included in the overarching PEPPPSY study, where initially 7 patients dropped out due to predefined exclusion criteria and personal reasons. All patients who used the SM function between December 2022 and June 2023 were included in this branch of the study. Thus, a total of 38 patients and 4 HCPs were included in the analysis. For 1 patient, sociodemographic data cannot be reported due to missing values.

Patients ranged in age from 18 to ≥60 years, with a mean age of 46.36 (SD 14.61; median 44.5, IQR 34.5-64.5) years. The overall study population (N=116 patients) had a similar age distribution to our sample, with a mean age of 45.39 (SD 15.76) years. As statistics show, the age distribution of the patients participating in this study also largely matches the age distribution of the general patient population in POCs in Germany (30% of patients are aged between 19 and 45 years and 40% of patients are aged between 45 and 69 years) [35]. Of the 38 patients in this sample, 25 (66%) were female and 13 (34%) were male. In the overall PEPPPSY study population, the gender distribution was comparable (male: 45/116, 38.8% and female: 67/116, 57.8%). Of the 116 participants, 2 (1.7%) did not disclose their gender identity. All patients in our sample were White and spoke German as their first language. Of the 38 patients, 4 (11%) had a family background of migration. The overall study population was slightly more diverse, with 3.5% (4/116) non-White patients. Similar to our sample, 10.3% (12/116) of the patients in the total population had a personal or family migrant background. In addition, 4.3% (5/116) of the patients did not speak German as their first language. Regarding employment status, 32% (12/38) of the patients in the study sample were employed, while 50% (19/38) were retired. Of the 38 patients, 1 (3%) was unemployed and 5 (13%) were unable to work. One patient (1/38, 3%) did not disclose the employment status. The employment distribution in the total study population was similar, with 32.8% (38/116) employed. However, there was a slightly higher proportion of unemployed patients (10/116, 8.6%) and patients who were unable to work (21/116, 18%), while the proportion of retired patients was significantly lower (44/116, 38%).

Table 2 shows the distribution of *ICD-10* diagnoses for both the entire PEPPPSY study population and our sample. In the *ICD-10* classification system, the numerals F1 to F9 are assigned to represent distinct diagnostic categories. Each code is designated to specify a particular group of mental and behavioral disorders.

**Table 2:** Distribution of diagnoses according to the German version of the International Classification of Diseases, Tenth Revision (ICD-10) categories in the total population of the PEPPPSY (Piloting and Evaluation of Participatory Patient-Accessible Electronic Health Record in Psychiatry and Somatics) study (n=116) and the sample reported in this study (n=38).

*ICD-10* codes	Study population (PEPPPSY study), n (%)	Study population with SM^a^ use, n (%)
F1^b^: Mental and behavioral disorders due to psychoactive substance use	2 (1.72)	0 (0)
F2: Schizophrenia, schizotypal, delusional, and other nonmood psychotic disorders	12 (10.34)	5 (13.51)
F3: Mood (affective) disorders	60 (51.72)	17 (45.59)
F4: Anxiety, dissociative, stress-related, somatoform, and other nonpsychotic mental disorders	20 (17.24)	7 (18.92)
F5: Behavioral syndromes associated with physiological disturbances and physical factors	0 (0)	0 (0)
F6: Disorders of adult personality and behavior	16 (13.79)	5 (13.51)
F7: Intellectual disabilities	0 (0)	0 (0)
F8: Pervasive and specific developmental disorders	1 (0.86)	0 (0)
F9: Behavioral and emotional disorders with onset usually occurring in childhood and adolescence	2 (1.72)	1 (2.7)
Missing diagnosis	3 (2.59)	2 (5.41)

^a^SM: secure messaging.

^b^F1-F9 refer to diagnostic categories according to the *ICD-10* classification system, where each code represents a specific group of mental and behavioral disorders.

The diagnoses in our sample largely reflect those in the total study population, although the latter exhibits slightly greater diagnostic diversity. As statistics show, the diagnosis distribution of the patients participating in this study largely corresponds to the diagnosis distribution of the general patient population in POCs in Germany. Most patients in POCs are treated for psychotic disorders (*ICD-10* F2) and affective disorders (*ICD-10* F3). However, compared to the general population of German patients in POCs, substance-related disorders are significantly underrepresented in our sample [38].

### HCP Characteristics

All HCPs were male medical physicians trained in psychiatry and behavioral psychotherapy and provided both medical and psychotherapeutic treatment. Of 4 HCPs, 2 (50%) were senior physicians, and 2 (50%) were specialist physicians in psychiatry. Furthermore, 3 HCPs worked part time (50% positions), while 1 HCP held a full-time (100%) position in the POC. HCPs ranged in age from 38 to 56 (mean 45.25, SD 8.14) years. Their clinical experience ranged from 6 to 23 (mean 11.5, SD 7.85) years. Of the 4 HCPs, 2 (50%) stated that they had never shared their clinical notes with patients before the study. The other 2 HCPs, however, reported that they had already occasionally provided patients with (parts of) the clinical documentation, for example, by documenting together or providing printouts. Moreover, 71% of the specialists in psychiatry and psychotherapy working in Germany are aged > 50 years. This means that the HCP study population is somewhat younger than the average [34]. In addition, it is likely that male psychiatrists are significantly overrepresented in our sample compared to the overall population. In 2023, an equal number of new specialist titles in psychiatry and psychotherapy were awarded to women and men [39].

### Message Characteristics

**Figure 2:**
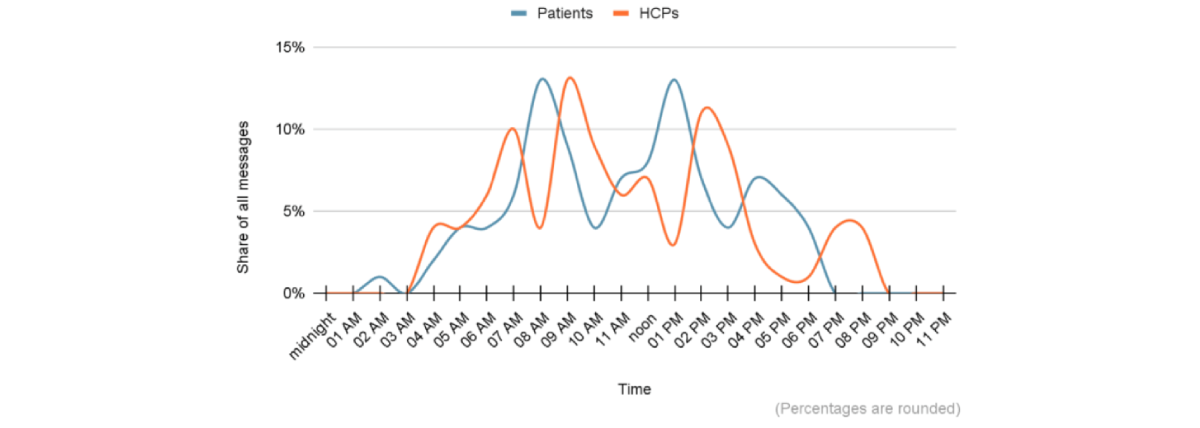
Use patterns among patients and health care professionals (HCPs) of the PEPPPSY (Piloting and Evaluation of Participatory Patient-Accessible Electronic Health Record in Psychiatry and Somatics) patient portal over time, showing the distribution of secure messages.

A total of 274 messages were analyzed: 61 (22.3%) initial clinical notes written by HCPs, 122 (44.5%) responses from patients, and 91 (33.2%) responses composed by HCPs in reaction to the patients’ messages. The number of messages sent by each patient ranged from 1 to 15, with an average of 4.16 and an SD of 3.42. The number of log-ins by patients to the web portal ranged from 1 to 42 (mean 10.78, SD 9.38). Most messages were sent by both patients and HCPs during the daytime. Nevertheless, there were also instances of messages being sent at night, at the earliest hours of the morning, and during the nighttime (Figure 2).

### Qualitative Results

All 274 messages were coded. Overall, the following four core functions and a number of subfunctions were identified: (1) reporting or feedback, (2) interpersonal, (3) intrapersonal, and (4) organizational. The presentation follows these core functions and is illustrated by example quotes from the messages of the study participants.

#### Organizational Function

All 3 types of messages (initial clinical notes, patients’ responses, and HCPs’ responses) were used to discuss organizational matters. These included matters pertaining to appointments and the issuing of formalities such as sick notes. Example quotes can be found in Table 3. Initial clinical notes contain only very few organizational matters. In their responses to initial clinical notes, patients contact HCPs with organizational requests, queries, and information regarding appointments (including via telephone), as well as the issuance of documents such as sick notes. Many patients are polite in their entries, while others express their concerns urgently or even demandingly. In their responses to patient entries, HCPs usually address the patients’ concerns in a friendly, supportive, and transparent manner. A significant proportion of organizational and scheduling requests are accommodated, but it is also clearly communicated when certain needs exceed the treatment scope or technical capabilities. HCPs frequently provided transparent information about their availability and absences, as well as alternative contact options (such as registering with the POC).

**Table 3:** Example quotes for organizational function, including initial clinical notes by health care professionals (HCPs), patients’ responses, and HCPs’ replies.

Message type	Example quotes
Initial clinical notes	“Please bring your treatment records from 2019 with you to your next appointment and also all your sick leave records from 2021, 2022 and 2023.” [HCP01]
Patients’ responses	“Please call me today as I urgently need a new prescription and need to discuss some things with you.” [PT01] “Could you please send me a scanned copy of the sick note to my email? I need it for work immediately.” [PT02] “Since my depressive symptoms have tended to worsen over the past week, I was wondering if there would be the possibility of obtaining a sick leave for the rest of the week?” [PT03]
HCPs’ responses	“This [sending scan to the employer] unfortunately doesn’t work. But I can assure you that your employer doesn’t need a scan directly. We will notify the employer automatically.” [HCP02] “If you need a sick leave for next week due to mental overload, feel free to drop by spontaneously. Unfortunately, I will only be available on Thursday and Friday next week—as those days are already fully booked and I’ll be on vacation for 3 weeks afterwards, you can just come by spontaneously and I’ll fit you in on Thursday/Friday in between.” [HCP03] “Thanks for the information, that’s enough for me. Then we won’t do another check-up for about 6 months. You don’t need to bring anything with you, I’ve already looked at it.” [HCP04]

#### Reporting and Feedback Function

##### Overview

Furthermore, all 3 message types were used to convey treatment-relevant information or to elicit feedback. Table 4 presents sample example quotes of the reporting and feedback functions found in each message type.

**Table 4:** Example quotes for reporting and feedback functions, including initial clinical notes by health care professionals (HCPs), patients’ responses, and HCPs’ replies.

Message type and reporting on		Example quotes
**Initial clinical notes**		
	Current situation	“Patient herself reports gradually increasing memory problems, especially forgetting appointments and conversations, probably said the same thing more often. The son reports that when the husband died in 2020, the things he had previously taken over no longer worked.” [HCP01] “Multiple pre-existing conditions, malfunctioning pituitary gland leading to increased urine production, requires self-administration of cortisone. His major issue is the lack of energy, along with constant sweating. Overall, there is excessive need for sleep. Additional problems include low mood and lack of drive.” [HCP02]
	Treatment	“Personal appointment: Midday dose of haloperidol made quite tired in the afternoon, stopped on his own. Quetiapine increased, short-term follow-up appointment.” [HCP04] “So it was discussed with the patient that it was very important to stand up for herself and to set boundaries. This is just as important for the family, for family members who have concerns. Discussed with the patient how she could do this with her family.” [HCP03]
**Patients’ responses**		
	Current situation	“At the moment, I am feeling very well, but I haven’t told you yet that I’ve had a high heart rate for years. My average is around 110.” [PT05] “I am under constant tension, stress, feeling unmotivated, irritable, and prone to outbursts. I just can’t seem to calm down anymore. My colleagues at work, who have a strong backbone, are experiencing the same thing. We have stomach and intestinal complaints in the office due to all the frustration because nothing changes. My migraine attacks and physical joint complaints have also increased again. I am experiencing insomnia despite taking sleeping pills.” [PT06] “My self-sacrifice began in my childhood, around the age of 10. I had to do everything perfectly and neatly in the family home with my mother’s changing partners. She also expected me to look after my younger siblings....I still suffer from this.” [PT06]
	Treatment	“The Haloperidol works very well in the morning. The evening medication is also right and important as it is.” [PT07] “I have tolerated it well so far, I also notice a slight improvement. However, the tension is still very high several times a day. Does it take a while for the medication to build up (as with antidepressants, for example)?” [PT08]
	Initial clinical notes	“Hypohedonia? A hint on whether that requires treatment would be very helpful for me, or if you’ve noticed this more often.” [PT09] “Very well summarized. Therapy sessions and subsequent reading help in working towards the goal.” [PT06] “Thank you for your note. Wouldn’t it also make sense to describe why I’ve been gradually tapering off the medication since summer, in consultation with my GP?” [PT10]
	Experiences with SM^a^	“I’ve also discovered the commenting feature now. You just need to click on the displayed entry—I should be more courageous.” [PT11] “I’m not sure if this [SM] is even meant for that purpose, but since I find it difficult to mention things/problems, etc., in the appointment, I just wanted to write something briefly here.” [PT12]
**HCPs’ responses**		
	Patients’ entries	“Thank you very much for your report, which is very helpful for further treatment decisions. I have read it and will incorporate it into the next appointment.” [HCP02] “Oh, you’re absolutely right! I fortunately noted it correctly in our medication module and corrected it here.” [HCP03]
	Treatment and current situation	“It’s indeed no problem to switch to Bupropion or Moclobemide. Would you like to come in on Monday for that?” [HCP02] “Firstly, bisoprolol has fewer side effects than metoprolol and can do the same thing, which is good. Secondly, you are welcome to submit the documents, but I cannot promise that I will be able to process them before Christmas. It often makes sense to clarify open questions together. In any case, it would make sense to add half an hour to the December appointment. Please let me know when you hand in your documents at the registration desk.” [HCP01]
	Experiences with SM	“Please feel free to continue documenting here if you’d like. It is helpful for our next appointment.” [HCP02] “By the way, the issue of comments appearing more than once is more of a technical problem; it’s happening to others as well.” [HCP01] “I’m sorry I’m just now replying, unfortunately the programme didn’t show correctly that you had written something. Do you have any questions? If so, please make an appointment with me. Kind regards” [HCP03]

^a^SM: secure messaging.

##### Initial Clinical Notes

In initial clinical notes of the HCPs, the reporting function predominated. HCPs described the current psychological, somatic, and social condition through the patient’s medical and sociobiographical history, previous treatments, current symptoms, social and occupational situation, and acute stressors in daily life. This diverse information enables a holistic understanding of the patient’s current concerns and sheds light on the reciprocal interactions between somatic health, mental health, and environmental factors. Furthermore, HCPs documented information on previous, current, and planned therapeutic and psychotherapeutic and medical treatments, usually considering the patient’s wishes. HCPs seem to take great care to report information in neutral, nonjudgmental language and usually do not address patients directly (refer to the Interpersonal Function section for exceptions). Although HCPs mention their own impressions of the patient’s status in the messages, they mostly focus on the patient’s own perception and refer to it in direct quotations or indirect speech (using phrases such as “Patient summarizes...” and “Patient reports...”).

##### Patients’ Responses

In their responses, patients very frequently provided specific feedback on the course of treatment, particularly regarding the tolerability of the medication. While some patients conveyed their experiences in an objective manner or expressed agreement with the practitioners’ approach, others voiced doubts, concerns, or hopes regarding their treatment. Specifically, when patients harbored doubts about their medical treatment or felt dissatisfied with side effects or the absence of improvement, they turned to the HCPs with direct questions. Subsequently, the patient would inquire about potential adjustments to their medication or seek clarification on its mechanism of action. Furthermore, patients supplement the information documented by HCPs in the initial clinical notes and provide feedback on changes to their current psychological and somatic condition, as well as on changes or stressors in their social or professional environment. In addition, some patients offer further insight into their personal biographical history, which seems pertinent to the development of their mental health condition and its (psychotherapeutic) treatment. For some patients, reporting appears to have a relieving effect, allowing them to express their distress instead of carrying it alone. Others use the entries as a sort of diary for self-motivation and empowerment. This possibility to relieve oneself appears to be of particular significance for patients with severe mental health conditions and is perceived as beneficial for their mental health. In addition to stressors and deteriorations, small improvements and joyful moments are also shared with the HCPs. A novel aspect is that patients provide explicit feedback on the initial treatment documentation by their HCPs as well as on the SM web application. Regarding SM, issues pertaining to usability and accessibility (eg, technical difficulties, errors, and features) are typically addressed, particularly when patients have cognitive impairments. However, patients also refer to the initial documentation in terms of content; they pose questions, express rejections and suggestions for corrections, and also offer praise and agreement. Furthermore, patients reassure themselves with their HCPs that they are allowed to use the web application for writing entries.

##### HCPs’ Responses

In addition, HCPs reference the entries of the patients in their responses; they express gratitude for composing the comments, ascribe significance to them, and encourage further use of the function. Furthermore, they rectify any potential errors in their initial clinical notes and pose content-related questions regarding the entries of the patients and their current situation. The responses to patients’ questions on further treatment are of a high standard. Patients receive timely appointment offers, psychoeducational explanations, treatment recommendations, information on dealing with emergencies, and collaborations with other HCPs. In the event of technical difficulties with the patient portal, HCPs reassure patients and clarify that the problems are not the patients’ fault but rather technical errors in the system. Overall, it is noticeable that the HCPs patiently and empathetically address every concern of the patients.

#### Interpersonal Function

##### Overview

In most entries from both HCPs and patients, explicit relationship messages can be identified (Table 5). In addition, the entries also contain a multitude of implicit relationship messages. For example, the predominantly empathetic and compassionate composition of the entries already indicates a certain fundamental interpersonal character in almost all entries. At the same time, they are kept very professional and appropriately focused. Furthermore, the salutation and farewell messages (which range from formal to friendly and from distant to familiar) contain relevant relational aspects. Interpersonal aspects can thus be found in almost all other categories in one way or another. However, because interpersonal aspects are both distinct and highly important, they are discussed separately in this section.

**Table 5:** Example quotes for interpersonal function, including initial clinical notes by health care professionals (HCPs), patients’ responses, and HCPs’ replies.

Message type	Example quotes
Initial clinical notes	“Discussed with patient that if condition worsens, sick leave is possible at any time. Should take good care of herself, set boundaries if something becomes too much, listen to herself.” [PT03] “Please observe in the next period: Sweating? Improvement in daytime drive/sleep needs? Erectile problems?” [HCP02]
Patients’ responses	“Thank you for listening and for these messages here.” [PT10] “By the way, I’m terribly afraid that I’m bothering you, that you might think I’m stupid... That’s why I’ve been ‘writing’ this here for almost 2 months.” [PT12]
HCPs’ responses	“I’m sorry to hear that the group therapy didn’t go as you hoped—if I understand correctly. I can only say that I am sure both the therapist and the fellow patients should (and will) have the utmost understanding that not everything always goes as planned in such a group. I want to encourage you and say that you are not to blame. I believe whatever has been/is okay. I hope you can put these thoughts aside. If you feel worse, please come to the outpatient clinic.” [HCP02] “I hope you were able to regain some stability in this difficult phase of life. Feel free to schedule another appointment if needed. I wish you much strength!”[HCP03]

##### Initial Clinical Notes

Given that HCPs primarily focus on reporting in the initial clinical notes, the interpersonal function is only subtly present. Nevertheless, a few HCPs include direct questions to patients (regarding the course and tolerability of medications) or “homework” in the initial clinical notes, thus initiating an active exchange.

##### Patients’ Responses

In their responses, patients convey relationship messages in multifaceted ways. A common theme in patients’ responses is gratitude for the messages, responses, and support provided by HCPs. Furthermore, many HCPs also receive positive sentiments, such as well-wishes from patients. Both gratitude and good wishes can contribute to the strengthening of the therapeutic relationship. Similarly, patients use the entries to seek reassurance, unload burdens, or address sensitive topics that they may be reluctant to discuss in person. In general, patients write their entries in a friendly and appropriate manner, which is almost professional and unobtrusive. However, in other responses, their distress becomes more apparent and is (more or less) implicitly directed as an appeal to the HCPs.

##### HCPs’ Responses

The responses of HCPs very frequently include messages and offers related to the establishment and maintenance of the therapeutic relationships. HCPs strengthen, encourage, and reassure patients through their compassionate and empathetic messages. As frequently described, HCPs always address the wishes and questions of patients in a patient and considerate manner. Similar to the patients, HCPs promote a good therapeutic relationship by expressing gratitude and good wishes to the patients and repeatedly encourage further exchange by posing specific questions. Furthermore, HCPs endeavor to foster transparency regarding their treatment and documentation practices and are not afraid to use self-disclosures. They respond authentically to patients, expressing their joy over (small) progress and demonstrating genuine understanding and compassion in the face of setbacks.

#### Intrapersonal Function

In the initial clinical notes, and particularly in the responses of the patients, an intrapersonal function of the SMS text messages could be identified. Both HCPs and patients used their messages as a direct or more indirect “note to themselves.” Table 6 provides example quotes. HCPs occasionally record information in the initial clinical notes that serve as reminders for their next appointment, specifying what they should pay attention to. This information is typically brief and documented only with keywords. These messages often contain questions and naturally also have an interpersonal character, as previously discussed. Patients use their entries to reflect on their treatment, recap their insights, and provide themselves with words of encouragement.

**Table 6:** Example quotes for intrapersonal function, including initial clinical notes by health care professionals (HCPs) and patient’s response.

Message type	Example quotes
Initial clinical notes	“For the next appointment: Plan blood sample, document weight.” [HCP04] “Alternative medication: Milnacipran? Bupropion?” [HCP02]
Patient’s response	“Realization: Patience pays off—miracles are possible...Do not be intimidated, continue exercising, overcome fear of driving, and overall work on self-confidence.” [PT11]

## Discussion

### Principal Findings

In the clinical text messages of patients affected by mental health conditions and HCPs at outpatient clinics in a rural area of Brandenburg, 4 main functions have been identified: organization, reporting and feedback, interpersonal uses, and intrapersonal uses. Despite the pervasive early fears about using SM with patients with psychiatric disorders, the interactions between HCPs and patients via SM were overwhelmingly positive in this study. This discrepancy between the anticipated and actual outcomes of SM is similarly reflected in other current studies [14]. The predominantly patient-centered, respectful, and friendly composition of the entries aligns with other empirical evidence [40,41].

Communication via SM poses challenges not only for patients but also for HCPs. The possible multitude of messages that must be managed alongside the already tightly organized treatment routine can be overwhelming [22,42]. Given that patients’ responses via patient portals could be read and answered at any time, including weekends or after work hours, patients may be under the impression that HCPs are available at all times via SM and will respond promptly to any queries [43,44]. Thus, HCPs must be particularly mindful of their own boundaries [44]. In our experience, attempts to facilitate boundary setting by shutting down the patient portal on weekends caused more confusion than relief among both HCPs and patients. In our study as well as those by others, HCPs endeavor to respond to queries in a timely manner, yet their work demands often preclude them from doing so. Consequently, responses may be delayed or occur outside of their scheduled clinic hours [45,46]. Furthermore, delays may occur due to technical issues, such as messages not being displayed immediately [5,23,42]. On the basis of the use patterns of patients in this study, it can be assumed that patients primarily contact their HCPs during main working hours. Nevertheless, web portals should provide clear indications that HCPs will not read and respond to entries immediately, emphasizing that it is an asynchronous communication process. This is particularly pertinent in the context of acute crises, such as suicidal intentions [47,48]. In the event of a crisis, it is imperative that the web portal provides clear and readily accessible information on emergency numbers. No such emergency occurred in this study. However, in emerging crises, HCPs provided relevant emergency contact information to patients via SM. As the web application develops, it may be possible to implement an automated search function that identifies messages containing specific keywords (such as suicidal, suicide, and self-harm) using artificial intelligence technology [49]. This then could trigger emergency responses or display the appropriate emergency contact numbers.

As the results of this study show, both HCPs and patients use SM for organizational matters (organizational function). Communication via the patient portal can offer a simple and fast alternative to telephone or in-person coordination in the POC, which is often associated with long waiting times. However, HCPs must then reliably and consistently review and respond to incoming messages, which could lead to an additional workload. Besides the patients, only medical personnel had access to our web application. However, nonmedical practice assistants could also receive access to the patient portal to assist and support with organizational matters, thereby relieving the workload of HCPs. As is shown in our study as well as in those by others, this seems to be particularly useful for dealing with organizational issues, such as scheduling appointments via patient portals, which was frequently observed in the SM interactions [50].

SM offers patients the opportunity to provide feedback and ask questions related to their HCPs’ clinical notes (reporting and feedback function). This allows patients to better understand their condition and treatment while also identifying potential errors and clarifying misunderstandings. Such processes were frequently observed in the message exchanges analyzed in this study. In line with previous research, it can be assumed that SM enhances treatment transparency and strengthen patient engagement in their own care.

Alongside the challenges it presents, communication via SM offers several benefits. In alignment with other studies, our research findings indicate that enhancing the therapeutic communication represents one of the most significant potential benefits (interpersonal factor) [44,48]. Some patients may feel more comfortable discussing sensitive topics through this mediated format than in direct contact with HCPs, often because of feelings of shame. In outpatient psychiatric care with brief and infrequent therapeutic contacts, patients often encounter difficulties in developing sufficient trust to openly discuss shameful or stigmatizing topics with their HCPs [51]. The interpersonal function of communication via SM facilitates more frequent and closer contact with HCPs, thereby possibly strengthening the therapeutic contact [52]. Moreover, HCPs sometimes seem to act as auxiliary egos for patients, offering support in important functions of the self, such as holding, containing, marking emotions, and self-reflecting [53]. However, it also becomes evident that in nearly every entry—often in just 1 sentence—many of these functions simultaneously come into play, interact, and overlap. On a communicative level, a sentence can possess diverse functions. Thus, it can be presumed that already established models of multifaceted communication, such as those by von Thun [54], also apply to communication mediated via SM [54]. Although this communication is conducted through digital and written channels, it is not merely a matter of exchanging factual and technical information. Instead, it is an intricate process of communication between HCPs and patients, with similarities to nondigital forms of communication. Conversely, the absence of nonverbal cues and the lack of immediacy inherent to digital communication via SM present HCPs with a set of novel challenges [42]. These insights must be considered when composing clinical notes and responses. It requires appropriate training and guidelines so that HCPs can have a certain level of expertise and confidence in this area [14,23,47].

Furthermore, SM offers patients the opportunity to engage with the content of their therapeutic sessions even after their appointments have ended (intrapersonal function). Some HCPs assign dedicated “homework” to patients, while other patients describe how reading the initial clinical notes and subsequent SM has helped them continue reflecting on the session’s content beyond the face-to-face therapy. This suggests that therapeutic interactions may extend into the digital space through SM.

Regarding the technical realization of SM in Germany, it can be stated that the German digital health agency “gematik” has briefly certified 7 different SM apps—so-called telematic infrastructure messengers, which all communicate using the matrix protocol via the German health data infrastructure [55]. The telematic infrastructure messenger currently supports communication only between HCPs, with patient-to-HCP communication planned for a future update. The patient portals enabled by the Hospital Future Act, which in turn should allow asynchronous, secure communication between patients treated by a hospital and their HCPs, are still used in only a few cases and have not yet met expectations. In this respect, the intervention of a patient portal with open notes and text-based asynchronous patient-HCP communication, as tested here, will only be possible under study conditions in the near future, at least in Germany [55].

### Limitations

The explorative nature of the study and its qualitative approach, while providing in-depth insights, may not be generalizable to the broader population. Furthermore, the sample was drawn from 3 POCs in a rural region, which may not represent the diversity of patients in different (eg, more metropolitan) geographical locations or other health care settings, such as the somatic field. In addition, no validated instruments were used to assess the digital and technical literacy of patients and HCPs. As a result, the study could not account for varying levels of technology and digital literacy among patients, which could have led to the exclusion of patients with lower technology or digital literacy. In contrast, our experience showed that even patients with limited digital skills were willing to participate in the study, though they required increased support and guidance from the study team. To foster the engagement of patient SM in routine care, trained nonmedical professionals (so-called “digital navigators”) who assist patients and HCPs in using the technology, thereby alleviating the burden on the treatment team, could be a promising option [56]. The health literacy and overall communication skills of the patients were also not specifically assessed, which may represent a limiting factor.

Furthermore, a potential gatekeeper effect should be considered, given that HCPs were responsible for patient recruitment in the PEPPPSY study [57,58]. However, all HCPs received comprehensive training on the eligibility criteria in advance and included a diverse range of patients with various psychiatric disorders and varying levels of digital literacy. Moreover, a potential self-selection bias should be considered. Patients who opt to use SM might be more engaged or proactive about their health care, whereas those who do not use the messaging feature might have different, unstudied needs or barriers. As nonusers of SM were not included, conclusions drawn from this study may primarily reflect the experiences of more engaged or technologically adept patients. In addition, by excluding patients with acute risk factors and significant cognitive impairments, the study might overlook the perspectives of those who are most vulnerable and might have unique challenges or needs related to the use of patient portals. Further research should prioritize the implementation of SM within these patient populations. In this study, SM was analyzed exclusively in the context of open medical record access. Future research could explore the use of SM in broader or alternative contexts.

### Conclusions

The study demonstrated the multifaceted nature of communication through SM in psychiatric outpatient care of patients affected by severe mental health conditions, with messages serving multiple purposes simultaneously. The complex communication via SM resembles traditional forms of communication while presenting unique challenges and opportunities. In addition to the necessity for training and guidelines for HCPs and personal assistance for patients to navigate digital communication effectively, structural adjustments in workflow are essential to manage the increased communication load and maintain professional boundaries.

Despite these challenges, SM offers notable benefits, including enhanced therapeutic contact and increased accessibility to care. However, further research is needed to ensure the meaningful use and responsible implementation of SM in health care, with particular attention to ethical, legal, and practical considerations.

## Appendix

Multimedia Appendix 1 COREQ (Consolidated Criteria for Reporting Qualitative Research) checklist.

## Abbreviations

COREQ: Consolidated Criteria for Reporting Qualitative Research
EHR: electronic health record
HCP: health care professional
ICD-10: International Classification of Diseases, Tenth Revision
PEPPPSY: Piloting and Evaluation of Participatory Patient-Accessible Electronic Health Record in Psychiatry and Somatics
POC: psychiatric outpatient clinic
SM: secure messaging

## Data Availability

The datasets generated or analyzed during this study are available from the corresponding author on reasonable request.
